# Expertise in die Fläche bringen: Analyse der Covid-19-Telekonsile und szenariobasierte Handlungsempfehlungen

**DOI:** 10.1365/s40702-022-00920-6

**Published:** 2022-10-31

**Authors:** Florian Neft, Karolin Eva Kappler, Sandra Maria Dohmen, Christian Juhra, Kathrin Sperling, Daniela Bause, Stefan Smolnik

**Affiliations:** 1 FernUniversität in Hagen, Hagen, Deutschland; 2 Katholische Hochschule Nordrhein-Westfalen, Köln, Deutschland; 3 Universitätsklinikum Aachen, Aachen, Deutschland; 4 Universitätsklinikum Münster, Münster, Deutschland; 5 Münster, Deutschland

**Keywords:** Covid-19, Technologien, Telekonsile, Telemedizin, Covid-19, Technology, Teleconsultations, Telemedicine

## Abstract

Das Auftreten der unbekannten Krankheit Covid-19 und die steigenden Covid-19-Fallzahlen stellten das Gesundheitswesen vor Herausforderungen. Die Häuser der Allgemeinversorgung waren bis dato größtenteils nicht mit der Behandlung eines schweren Lungenversagens vertraut und so musste dieses Wissen aus den Expertenzentren in die Häuser der Allgemeinversorgung transferiert werden. Dazu wurden im Rahmen der Vorstufe des Virtuellen Krankenhauses Nordrhein-Westfalen (VKh.NRW) Telekonsile genutzt. Sie finden zwischen zwei Ärzten der Intensivmedizin statt, stellen ortsunabhängig Expertenwissen bereit und erhöhen so die Behandlungsqualität sowie -effizienz. In der Evaluation dieser Telekonsile zeigen sich unterschiedliche Nutzungsszenarien. Während zu Beginn größtenteils allgemeine Diagnose- und Therapiewege von Interesse waren, kamen später detailliertere Fragen auf, für die unter anderem ein Pharmakologe notwendig war. Dieser Anwendungsfall bedarf mehr explizites Wissen, Patienteninformationen und damit erhöhte Technologieanforderungen. Im Rahmen der technischen Infrastruktur zeigen sich allerdings Barrieren. Daher wird evaluiert, welches Wissen in den unterschiedlichen Anwendungsfällen der Telekonsile ausgetauscht wird und welche technologischen Voraussetzungen erfüllt sein müssen, damit Telekonsile stärker zur Verwendung kommen und das Gesundheitswesen gleichermaßen entlasten und verbessern.

## Abstract (en)

The emergence of the unknown disease COVID-19 and the increasing number of COVID-19 cases challenged the health system. General, standard care hospitals were largely unfamiliar with the treatment of severe respiratory failure up to then, and so this knowledge had to be transferred from university hospitals to standard care ones. Therefore, intensive care physicians within the preliminary stage of the virtual hospital North Rhine-Westphalia (VKh.NRW) used teleconsultations. Teleconsultations occur between two intensive care physicians, make expertise anywhere available and increase treatment quality and efficiency. The evaluation of these teleconsultations shows different scenarios. Whereas initially, the enquirers largely addressed general diagnostic and therapy question regarding the COVID-19 treatment, they arose more detailed questions later, necessitating a specialist, such as a pharmacologist. This scenario demands more explicit knowledge, and, thus, increased technology requirements. Barriers appear in the context of this technical infrastructure. Therefore, we evaluate which knowledge is transferred within different teleconsultation scenarios and which technology requirements must be fulfilled to increase the use of teleconsultation and, thus, improve the health system.

## Die Herausforderungen der neuartigen Krankheit Covid-19

Telemedizinische Anwendungen werden in den letzten Jahren vermehrt eingesetzt (Haleem et al. 2021). Neben den Möglichkeiten, die Behandlung und die Kommunikation zwischen Arzt und Patient zu digitalisieren, existieren weitere Verwendungsformen wie der interdisziplinäre Austausch zwischen zwei Ärzten im Rahmen von Telekonsilen (Guinemer et al. 2021; Marx et al. 2022). Durch aufkommende technologische Möglichkeiten vergrößert sich auch das Spektrum der Telemedizin (Haleem et al. 2021). So können Patienten bei Telekonsilen zwischen zwei Ärzten, mithilfe von digitalen Visitenwagen, in die Notebook, Kamera und Mikrofon integriert sind, direkt am Bett versorgt werden. Dies ist ein Vorteil, da bereits kurze Transporte von kritisch erkrankten Patienten hohe Risiken mit sich bringen (Dohmen et al. 2022). Weiterhin bietet die Telemedizin den Vorteil, Expertenbehandlungen auch in Krankenhäusern anzubieten, die über keine entsprechend spezialisierten Fachärzte wie Infektiologen oder andere Fachdisziplinen verfügen (Marx et al. 2022). Damit ermöglicht die Telemedizin die ortsunabhängige Diagnose und Therapie des Patienten und die Vermeidung der Verlegung in ein Universitätsklinikum (Haleem et al. 2021). Dies hat sowohl auf das behandelnde Krankenhaus als auch auf das Universitätsklinikum einen positiven Effekt, da die Patientenbehandlung optimiert, die verfügbaren Mittel besser genutzt und nicht wertschöpfende Prozessschritte wie Transporte reduziert werden (Haleem et al. 2021).

Trotz dieser Potenziale ist die Nutzung der Telemedizin durch die vorhandene technologische Infrastruktur gehemmt (Lupton und Maslen 2017). Während für ein Telekonsil zwischen zwei Ärzten ein Notebook mit Videokonferenzsoftware ausreichend sein kann, wird für gewisse erweiterte telemedizinische Vorhaben moderne Sensorik benötigt (Guinemer et al. 2021; Dohmen et al. 2022). Diese wachsenden Technologieanforderungen stellen einen Großteil der deutschen Kliniken vor Herausforderungen, denn die notwendige Infrastruktur wie eine stabile WLAN-Verbindung in jedem Patientenzimmer sind nicht immer gegeben. Dadurch werden Optionen wie die Überwachung des Patienten mithilfe von Kameras oder die lückenlose Übertragung seiner Vitalparameter beeinträchtigt (Neft et al. 2021).

Neben diesen technologischen existieren weitere Herausforderungen, welche die Akzeptanz telemedizinischer Lösungen im Gesundheitswesen im Vergleich zu anderen Branchen stärker einschränken (Haleem et al. 2021). Gesundheitsdaten sind sensible Informationen. Daher müssen telemedizinische Anwendungen die Privatsphäre der Daten gewährleisten (Haleem et al. 2021). Gleichzeitig besitzen die behandelnden Ärzte eines Patienten vom Hausarzt bis zum Krankenhausmediziner unterschiedliche Zusatzqualifikationen. Diese führen zu differenzierten Wissenssilos. Um den Patienten bestmöglich zu behandeln und diese Wissensfragmentierung aufzulösen, ist der interorganisationale Austausch mehrerer Ärzte notwendig. Es existieren bereits zahlreiche Forschungsartikel, die den intraorganisationalen Wissensaustausch untersuchen, beispielsweise im Rahmen von Krankenhausinformationssystemen (Chen et al. 2019). Allerdings ist die Forschung zum interorganisationalen Wissensaustausch im Gesundheitswesen aufgrund von ungeklärten Fragen zur Privatsphäre oder fehlenden technologischen Möglichkeiten eingeschränkt und weist daher eine Forschungslücke auf (Chen et al. 2019).

Der interorganisationale Wissensaustausch ist essenziell, um neuartige Krankheiten wie Covid-19 zu behandeln (Peine et al. 2020). Zur Therapie von Covid-19 wurde zunächst Wissen über die Krankheitsentstehung und den -verlauf gesammelt (Haßmann 2020). Zu Beginn des Auftretens schwerer Covid-19-Fälle wurden die Patienten hauptsächlich in Universitätskliniken versorgt (Haßmann 2020). Nachdem die Fallzahlen stiegen, nahmen auch die Kliniken der Allgemeinversorgung Covid-19-Patienten auf. Allerdings hatten sie bis zu diesem Zeitpunkt kaum Erfahrung mit der Covid-19-Behandlung (Haßmann 2020). Daher wurden Wege gesucht, die Erfahrungen und das Wissen der Universitätsmediziner den Häusern der Allgemeinversorgung zur Verfügung zu stellen. Die Vorstufe des virtuellen Krankenhauses Nordrhein-Westfalen (VKh.NRW) hat zu diesem Zweck eine Plattform etabliert, die Mediziner zur Durchführung von Telekonsilen nutzen können. Dabei werden Fragen zur Behandlung von Covid-19-Patienten gemeinsam mit einem Universitätsmediziner geklärt. Diese Covid-19-Telekonsile stellen daher ein optimales Nutzungsbeispiel der Telemedizin dar, das zeigt, wie technologische Barrieren überwunden werden und interorganisationaler Wissensaustausch stattfinden kann. Demzufolge ist das Ziel dieses Beitrags, die dargestellten Forschungslücken zum Wissensaustausch und die Voraussetzungen der technologischen Infrastruktur für die Krankenhäuser zu evaluieren:

### Forschungsziel

*Evaluation des Wissensaustauschs und der technologischen Voraussetzungen der Covid-19-Telekonsile.*

Dieses Forschungsziel wird anhand qualitativer Interviews mit 22 Teilnehmern der Covid-19-Telekonsile des VKh.NRW beantwortet. Dabei wird gezeigt, inwieweit im Rahmen der Telekonsile unterschiedliche Wissensformen nach Nonaka et al. (2000) ausgetauscht werden. Diese Wissensformen sind je nach Zweck unterschiedlich ausgeprägt und formen daher die drei Szenarien „Expertenkonsile ohne Fallbezug“, „Expertenkonsile mit Fallbezug“ und „Rekonsile“. Weiterhin werden die technologischen Voraussetzungen identifiziert, welche für das jeweilige Szenario erfüllt sein müssen. Der Beitrag schließt mit einer Empfehlung, wie die Nutzung von Telekonsilen zukünftig erhöht werden kann.

## Technologien der Telekonsile

Im klinischen Alltag existieren diverse Formen, Wissen intraorganisational auszutauschen. Mediziner nutzen unter anderem Visiten, um sich gegenseitig, regelmäßig zu einem Patienten zu beraten und die weitere Behandlung zu evaluieren und damit zu optimieren. Dieser Wissensaustausch rettet Menschenleben (Marx et al. 2022). Die auf der Intensivstation behandelten Covid-19-Patienten, die im Rahmen der Covid-19-Telekonsile thematisiert wurden, waren schwerstkrank. Um die Häuser der Allgemeinversorgung zu befähigen, diese Patienten bestmöglich zu therapieren, musste die Expertise der Universitätskliniken virtuell vermittelt werden. In diesem Kontext umfasst die Telemedizin jegliche Verwendungsformen von Informationstechnologien zur räumlich distanzierten Versorgung mit dem Zweck der Diagnose, Therapie oder Rehabilitation. Eine Subkategorie sind die Telekonsile, deren unterschiedliche Anwendungsfälle in Tab. 1 dargestellt sind (Greenhalgh et al. 2020; Haßmann 2020).

**Tab. 1:** Formen von Telekonsilen

Form	Zweck	Technologien	Beziehung	Quelle
Ambulant	Diagnose einer Krankheit: Patient adressiert eine medizinische Fragestellung an einen Arzt	Telefon, Videokommunikationssoftware, Notebook, Kamera, mobiles Netzwerk	Arzt und Arzt, Arzt und Patient	Greenhalgh et al. (2020); Koncz et al. (2019)
	Diagnose einer Krankheit: Beratung zwischen medizinischem Personal (z. B. Telerettungswagen) zur Identifikation akuter Erkrankungen (z. B. Schlaganfall)			
Stationär	Gegenseitig Beratung zu einem stationären Patienten zwischen Medizinern	Telefon, Videokommunikationssoftware, Notebook, Kamera	Arzt und Arzt (und Patient)	Haßmann (2020); Dohmen et al. (2022)

Die Telekonsile sind definiert als die Beratung zweier Ärzte oder eines Arztes und eines Patienten zur Untersuchung einer medizinischen Fragestellung. Dieser digital gestützte Wissensaustausch befähigt Mediziner, optimale Entscheidungen für den Patienten zu treffen. Anders als die Visite findet ein Konsil nicht regelhaft statt, sondern wird explizit angefragt. Tab. 1 zeigt unterschiedliche Anwendungsfälle der Telekonsile, beispielsweise zur Diagnose oder zur gegenseitigen Beratung der weiteren Behandlung. Je nach Zweck existiert ein unterschiedlicher Informations- und damit einhergehend Technologiebedarf für das jeweilige Telekonsil. Während in manchen Fällen Telefon oder Notebook ausreichend für die Teilnahme am Telekonsil sind, werden für andere Formen Kamera, WLAN-Verbindung am Patientenbett und Breitbandnetzwerk benötigt (Greenhalgh et al. 2020; Koncz et al. 2019). Einige Anforderungen wie die technologische Ausstattung in Form der vorhandenen Geräte kann das Krankenhaus beeinflussen, während andere Komponenten wie ein stabiles Internetnetz regional bedingt sind und nicht durch das Krankenhaus veränderbar sind (Forge und Vu 2020). In diesem Zusammenhang zeigen Studien die Vorteile der Nutzung von 5G-Netzwerken, beispielsweise in der Teleradiologie in China. Während diese Netzwerke dort bereits etabliert sind, werden für andere Länder Implementierungsstrategien entwickelt (Forge und Vu 2020). Dieses Beispiel zeigt den Zusammenhang zwischen technologischen Voraussetzungen und Anwendungsformen. Um diesen Zusammenhang für die Telekonsile zu prüfen, stellen wir die folgenden Forschungsfragen:

1. *Welche technologischen Voraussetzungen müssen Telekonsile zum Wissensaustausch erfüllen?*
2. *Inwiefern stellen Krankenhäuser diese technologischen Anforderungen zur Verfügung?*

Je nach Telekonsil werden unterschiedliche Wissensformen weitergeben. Einerseits wird explizites Wissen geteilt, beispielsweise in Form von konkret formulierten Behandlungsempfehlungen, andererseits wird implizites Wissen wie Gedanken oder Behandlungstipps ausgetauscht (Nonaka et al. 2000). Das differenzierte Interesse nach implizitem oder explizitem Wissen bildet unterschiedliche Szenarien. Diese werden evaluiert, um szenariobasierte Anforderungen zu identifizieren.

## Vorgehen

**Abb. 1:**
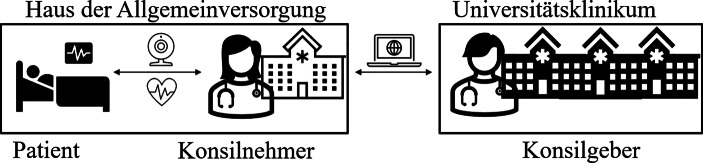
Telekonsile im Rahmen der Vorstufe des VKh.NRW

Die aufgestellten Forschungsfragen werden anhand der Ergebnisse einer qualitativen Interviewstudie beantwortet. Im Rahmen der Vorstufe des VKh.NRW fanden seit März 2020 insgesamt mehr als 3800 Covid-19-Telekonsile bei mehr als 600 Patienten in 41 Krankenhäusern statt. Das VKh.NRW ist eine, durch das Land NRW, finanzierte Plattform zum Austausch für Mediziner. Die Vorstufe des VKh.NRW fokussierte sich auf die Expertise von Ärzten, die in der Intensivmedizin tätig sind und das Krankheitsbild Covid-19. Demzufolge haben die beiden Universitätskliniken Aachen und Münster die insgesamt 41 Häuser der Allgemeinversorgung zu schwerstkranken, sich auf der Intensivstation befindlichen, und teilweise nicht ansprechbaren, Covid-19-Patienten beraten. Diese Konfiguration ist in Abb. 1 ersichtlich.

Der Fokus der dargestellten Telekonsile in Abb. 1 lag auf dem interorganisationalen Austausch zwischen den Kliniken. Um die differenzierten Anwendungsformen im Rahmen der Covid-19-Telekonsile zu identifizieren, wurden 22 Mediziner befragt. Der Großteil der Teilnehmer, insgesamt 17, stammt aus den Universitätskliniken (Konsilgeber), während fünf Mediziner aus den Häusern der Allgemeinversorgung (Konsilnehmer) teilnahmen. Unter den 17 Medizinern der Universitätskliniken ist ein Pharmakologe und ein Infektiologe. Die Stichprobe ist in Bezug auf das Geschlecht ausgewogen. Zusätzlich besitzen etwas mehr als die Hälfte der Befragten durch die Teilnahme an der Vorgängerstudie TELnet@NRW telemedizinische Vorerfahrung. Im Rahmen dieser Vorgängerstudie, die mit 159.424 Patienten eine der größten europäischen Telemedizinstudien darstellt, konsultierten Mediziner unter anderem zur Indikation Sepsis (Marx et al. 2022). Im Rahmen von TELnet@NRW fanden intensivmedizinische, regelmäßige Visiten statt, während das in dieser Forschung untersuchte Forschungsobjekt, das Telekonsil des VKh.NRW, explizit auf Anfrage erfolgt (Marx et al. 2022; Pecquet und Beckers 2021).

Nachdem die Ethikkommissionen beider Universitätskliniken die Interviewstudie im Oktober 2021 akzeptierten, wurden die Befragungen im Zeitraum von Oktober 2021 bis Januar 2022 durchgeführt. Da während dieser Periode die Zahl der Covid-19-Intensivpatienten stark anstieg, nahm auch die Belastung der Befragungsteilnehmer zu und bedingte die geringere Teilnahme der Konsilnehmer.

Die Forschungsfragen werden mithilfe eines Interviewleitfadens adressiert, der Fragen zum Aufbau, Transfer und der Aufbewahrung des ausgetauschten Wissens enthält. Weiterhin werden positive Aspekte und Verbesserungsmöglichkeiten der Covid-19-Telekonsile ermittelt. Die Interviews wurden aufgenommen und transkribiert. Mithilfe eines zuvor definierten Codebaums, der auf dem SECI-Modell nach Nonaka et al. (2000) basiert und sechs Codekategorien sowie insgesamt 70 Subcodes enthält, wurden die Transkripte codiert. Ein Beispiel für einen Code ist in Tab. 2 exemplarisch dargestellt:

**Tab. 2:** Im Rahmen der Telemedizin angewandte Technologien

Code	Subcode	Definition	Beispiel
*Problem*	Problem_Infrastruktur	Probleme aufgrund der technologischen Infrastruktur	„In einigen Krankenhäusern ist es immer noch so, dass das WLAN sehr, sehr schlecht funktioniert, und dann teilweise die Verbindung abbricht.“ – ID8
	Problem_Medienbruch	Probleme aufgrund von Medienbrüchen	„Was natürlich einen Datentransfer eher mühselig macht, wenn man das alles noch in Papierakten (…) erfragen, suchen muss.“ – ID13

Diese Codierung wurde durch einen zweiten Wissenschaftler validiert. Die Intercoderreliabilität beträgt 0,931, was auf eine hohe Übereinstimmung und damit hohe Reliabilität schließen lässt. Im Anschluss an die Codierung wurden gleich codierte Paragrafen im Rahmen der qualitativen Inhaltsanalyse gebündelt und zusammengefasst (Mayring und Fenzl 2019).

## Ergebnisse

Die Interviews zeigen einen unterschiedlichen Bedarf an explizitem und implizitem Wissen und damit mehrere Formen an Telekonsilen, die im Laufe der Covid-19-Wellen stärker oder schwächer nachgefragt wurden (ID18). Aus den Interviews lassen sich drei Anwendungsformen der Covid-19-Telekonsile ableiten:

1. Expertenkonsil ohne Fallbezug: Allgemeine Fragen zur Therapie, Diagnostik, etc. ohne konkreten Patientenbezug
2. Expertenkonsil mit Fallbezug: Fragen zu einem Patienten, die sich direkt klären lassen und kein weiteres Konsil benötigen
3. Rekonsil: Mehrmalig, aktiv angefragter Kontakt zu einem Patienten

Diese drei Szenarien differenzieren sich in Bezug auf den Zweck und die technologischen Anforderungen. Zu Beginn der Coronazeit wurden die Covid-19-Patienten primär in den Universitätsklinken behandelt (ID21). Die Häuser der Allgemeinversorgung hatten keine oder wenig Erfahrung (ID5). Dennoch mussten sie mit zunehmenden Infektionszahlen Covid-19-Patienten aufnehmen. In diesem Rahmen schildert eine Konsilnehmerin die Komplikationen bei der Behandlung der ersten Patienten (ID5). Aufgrund des geringen Behandlungswissens kontaktiert sie Kliniken in der Umgebung, die eine größere Anzahl an Coronapatienten betreuten (ID5). Demnach benötigt die Konsilnehmerin in diesem Fall implizites Wissen in Form praktischer Tipps und Erfahrungswissen zur Covid-19-Behandlung (ID5). Dieses Szenario bezeichnen wir als Expertenkonsil ohne Fallbezug, da allgemeine Fragen zur Diagnostik oder Therapie ohne konkreten Patientenbezug gestellt werden (ID5). Dazu werden Telefon oder Notebook als niederschwellige Möglichkeit genutzt, um die Universitätskliniken zu kontaktieren und das Erfahrungswissen der Universitätsmediziner abzuholen (ID5; ID10; ID11; ID12). Weiterhin berichten Konsilgeber von aufgenommenen Schulungsvideos, in denen Konsilnehmern bestimmte Praktiken beispielsweise zur Bauchlagerung vorgeführt werden (ID15).

Für patientenspezifische Fragen greifen die Konsilnehmer verstärkt auf das Notebook und eine zertifizierte Videokommunikationssoftware zurück (ID10). Diese Kategorie definieren wir als Expertenkonsile mit Fallbezug. Steigt der Bedarf des Informationsaustauschs, hat das Expertenkonsil mit Fallbezug den Vorteil, Daten zum Patienten in einer digitalen Fallakte festzuhalten (ID10; ID15). Dieses Expertenkonsil wird beispielsweise angefragt, wenn sich der Konsilnehmer vom Universitätsmediziner zusätzliche Impulse erhofft, die Behandlung zu optimieren (ID5; ID12; ID14). Daher ist das Expertenkonsil mit Fallbezug durch spezifische Fragen zu einem konkreten Patienten, die der Konsilnehmer nicht selbst beantworten kann, gekennzeichnet (ID5; ID12). Ein Beispiel für eine solche Anfrage ist folgender Abschnitt:

„Was würde man jetzt in diesem Schwangerschaftsstadium geben? Kann man ein Ersatzpräparat nehmen? Also da war (…) wirklich gut, dass man die Pharmakologie an seiner Seite hatte.“ – ID21

Diese individuellen Fragen benötigen teilweise den Rat von Fachexperten wie Infektiologen oder Pharmakologen, weshalb diese einmal wöchentlich an den Telekonsilen teilnehmen (ID6; ID19). Zusätzlich stimmen sich die Konsilgeber bei sehr spezifischen Fragestellungen klinikintern mit den Experten des jeweiligen Fachgebiets ab (ID3; ID22). So schildert ein konsilgebender Anästhesiologe ein gemeinsames Gespräch mit einem Chirurgen des Universitätsklinikums, um dem Konsilnehmer eine treffende Antwort zu geben (ID22). Innerhalb des Universitätsklinikums existiert häufig noch ein zweiter Kollege oder ein Oberarzt, der bei sehr spezifischen Fragen beratend unterstützen kann (ID6; ID18):

„Es gibt an der Uni immer (…) noch eine Ebene darüber oder noch einen anderen, der sich damit befasst.“ – ID18

Damit wird die gesamte Expertise des Universitätsklinikums für die Häuser der Allgemeinversorgung durch die Telekonsile zugänglich (ID1). Ein weiterer Grund für die Anfrage eines Expertenkonsils mit Fallbezug ist der Wunsch nach konkreten Behandlungs- oder Medikationsratschlägen für einen bestimmten Patienten (ID18; ID21; ID22). Dazu bedarf es eines detaillierten Patientenbildes, weshalb der Konsilgeber den Patienten bestmöglich sehen sollte, detaillierte Einblicke in seine Patientendaten haben und möglichst viel explizites Wissen in Form der bisherigen Dokumentation zum Krankheitsverlauf einsehen können sollte (ID8; ID21; ID22). Weiterhin wird dieses Expertenkonsil teilweise für Übernahmeanfragen von Patienten verwendet (ID12; ID22). Da die Häuser der Allgemeinversorgung keine Geräte und Personal für eine ECMO-Therapie zur Verfügung haben, diese aber bei besonders schweren Verläufen notwendig wird, werden mithilfe der Telekonsile Verlegungsanfragen gestellt (ID11):

„Ich sag mal der Vorteil lag für uns im Wesentlichen darin, dass wir kompetente Hilfe bekommen haben, zu entscheiden, wann wir denn einen Covid-Patienten an die Uni abgeben müssen (…). Im Wesentlichen dienten die Konsile immer der Frage: ‚Behandeln wir den Patienten bei uns weiter oder geben wir ihn an die Uni ab?‘“ – ID11

Dazu prüfen die Universitätsmediziner die patientenindividuellen Kriterien und beraten sich intern mit weiteren Fachspezialisten (ID22). Um in diesem Beispiel der Patientenverlegung oder auch um akut Antworten zu dem Expertenkonsil mit Fallbezug nachzureichen, nutzen die Konsilgeber häufig aufgrund der Schnelligkeit das Telefon (ID17; ID19; ID22). Insgesamt besteht in diesem Szenario im Vergleich zum Expertenkonsil ohne Fallbezug ein erhöhter Bedarf an explizitem Wissen und demzufolge auch zusätzlichen Technologien (ID8; ID21).

Werden weitere Behandlungen und Diagnosen seitens der Konsilgeber benötigt, um ein besseres Bild zum Patienten zu erhalten, wird der einzelne Patient teilweise wiederholt diskutiert (ID8). Die mehrmalige Durchsprache eines konkreten Patienten klassifizieren wir als Rekonsil (ID7; ID9; ID19). Die Rekonsile bezwecken die gemeinsame Diskussion des Patientenverlaufs (ID21). Demzufolge ist die Momentaufnahme wie im Expertenkonsil mit Fallbezug nicht ausreichend und die Menge des ausgetauschten, expliziten Wissens vergrößert sich (ID21). Bei diesen Rekonsilen präsentiert der Konsilnehmer den Patienten, dessen Verlauf und seine persönliche Einschätzung. Daraufhin stellt der Konsilgeber Fragen zur leitliniengerechten Behandlung (ID7; ID18). Zusätzlich werden in diesem Szenario Visitenwagen verwendet, sofern ein stabiles und ausreichendes WLAN-Netz existiert (ID9). Damit erhält der Konsilgeber ein besseres Bild vom Patienten (ID21). Sofern diese erhöhten Technologieanforderungen erfüllt werden, wird durch diese mehrmalige kollegiale Beratung zu einem Patienten, die Behandlung optimiert und Fehler vermieden (ID18; ID21). Tab. 3 fasst die drei aufgeführten Szenarien zusammen.

**Tab. 3:** Identifizierte Anwendungsformen und zugehörige Technologien

Szenario	1. Expertenkonsil ohne Fallbezug	2. Expertenkonsil mit Fallbezug	3. Rekonsil
*Definition*	- Allgemeine Fragen zu Covid-19	- Einmaliger Kontakt zu einem Patienten	- Mehrmaliger Kontakt zu demselben Patienten
	- Generalistisch, ohne konkreten Patientenbezug	- Detailliert, Momentaufnahme	- Detailliert, Patientenverlauf
		- Diskussion von Therapiemöglichkeiten bei komplexen Fällen	- Engmaschige Betreuung eines kritischen Patienten und des Krankheitsverlaufs
	- Erfahrungswissen zur Behandlung		
*Benötigte Technologien*	- Telefon	- Telefon	- Telefon
	- Notebook	- Notebook (inkl. Kamera)	- Notebook (inkl. Kamera)
		- Elektronische Fallakte	- Elektronische Fallakte
			- Erweiterte Befundmöglichkeiten
			- Kamera am Patientenbett

Neben der ersten Forschungsfrage zu den Szenarien, den ausgetauschten Wissensformen und den benötigten Technologien, die Tab. 3 beantwortet, wird innerhalb der zweiten Forschungsfrage die Verfügbarkeit dieser Technologien in den teilnehmenden Kliniken geprüft. Dabei zeigen sich signifikante Unterschiede. Mehrere regionale Kliniken schildern eine unzureichende Internetanbindung des jeweiligen Krankenhauses als Hemmnis für den vermehrten Einsatz der Telemedizin (ID11):

„Es gab schon mal technische Abstürze (…), die an den allgemeinen, digitalen Gegebenheiten in Deutschland liegen.“ – ID11

Zusätzlich gewährleistet die interne Krankenhausausstattung nicht in allen Kliniken eine ausreichende WLAN-Abdeckung in jedem Zimmer. Diese infrastrukturellen Probleme und weitere nicht performante Gerätschaften werden in mehreren Interviews hervorgehoben (ID11; ID12; ID18):

„Wir haben bis jetzt immer mit einer Kamera und einem Gerät gearbeitet (…). (…) da kommen wir immer wieder an unsere Grenzen: (…) ‚nach 30 min stürzen die Systeme ab (…).‘ Jedenfalls eigentlich ist die Telemedizin zurzeit bei uns, schlägt die um, in eine Telefonmedizin wieder, also wir haben gar nicht mit mehr die Möglichkeiten einer vernünftigen Bildgebung, vor Ort, am Patienten, weil die Systeme dann, die fangen dann an zu haken und (…) dann ist die Verbindung weg.“ – ID12

Experten- und insbesondere Rekonsile sind mit dieser technologischen Infrastruktur schwierig umsetzbar. Die Spezifikation der Telekonsile auf die Indikation Covid-19 erzeugt dahingehend weitere Einschränkungen in der Technologienutzung, denn durch die hohe Übertragbarkeit der Krankheit mussten jegliche Utensilien, die mit in das Patientenzimmer genommen werden, anschließend desinfiziert oder verworfen werden. Aufgrund der negativen Auswirkungen der Desinfektionsvorgänge auf die Haltbarkeit technischer Gegenstände, führt der Mediziner das Telekonsil meist in seinem Büro und nicht im Patientenzimmer durch. Konträr zu diesen Aussagen, nutzen andere Kliniken wiederum Televisitenwagen. Diese ermöglichen die Präsentation des Patienten mit einer Kamera in dessen Zimmer. Diese Visitenwagen erfüllen daher eine Voraussetzung für Experten- und Rekonsile. Eine weitere Notwendigkeit, der Zugriff auf die Fallakte, funktioniert in jedem Krankenhaus (ID10; ID11). Darüber hinaus besitzt lediglich eine der teilnehmenden Kliniken über ein entsprechendes Patientendatenmanagementsystem (PDMS), mithilfe dessen Patientendaten und Befunde einfacher geteilt werden können. Dieses PDMS ist vor allem für die Nachverfolgung des Krankheitsverlaufs und damit für die Rekonsile erforderlich. Die verstärkte Verwendung solcher Systeme vereinfacht dem Konsilgeber den Zugriff auf die Patientendaten. Somit könnte dieser den Fokus früher auf die eigentliche Frage des Konsilnehmers richten (ID2; ID22). Zusammenfassend zeigt sich damit für Expertenkonsile mit Fallbezug und Rekonsile Handlungsbedarf, weiteres technologisches Equipment in den Kliniken zu installieren und Patientendaten interorganisational schneller verfügbar zu machen, während für Expertenkonsile ohne Fallbezug die technologischen Anforderungen erfüllt sind.

## Diskussion und Ausblick

Die Literatur zeigt Anwendungsfälle, die über die drei geschilderten Szenarien hinausgehen. Beispielsweise wird im Kontext der Vorgängerstudie TELnet die Televisite beschrieben, bei der zu einem fest vereinbarten, regelmäßigen Termin Intensivpatienten besprochen werden (Marx et al. 2022). Weiterhin schildert Guinemer et al. (2021) die ortsunabhängige, zentrale Überwachung von Intensivpatienten. Diese Beispiele bedürfen die uneingeschränkte Verfügbarkeit der Internetanbindung am Patientenbett (Guinemer et al. 2021; Lupton und Maslen 2017). Nachdem bereits im Rahmen der Telekonsile diese Anforderungen in manchen Kliniken nicht gegeben sind, sind diese erweiterten telemedizinischen Möglichkeiten in solchen Kliniken noch nicht gewinnbringend. Sollte die Digitalisierung dieser Kliniken voranschreiten, bieten beispielsweise Televisiten die Möglichkeit, die Präsentation von Patienten zu üben oder das persönliche Wissen um Aspekte zu erweitern, die im Krankenhaus der Allgemeinversorgung nicht alltäglich sind (ID11; Marx et al. 2022). Zusätzlich besitzt die Televisite den Vorteil, die Behandlung zu kontrollieren und unwissentliche Behandlungsfehler zu identifizieren und folglich abzustellen (ID18; Marx et al. 2022). Dahingegen werden die, im Rahmen dieser Studie geschilderten, Telekonsile lediglich im Bedarfsfall angefragt (ID2; Pecquet und Beckers 2021). Insgesamt zeigt sich das Bedürfnis, die technologische Infrastruktur vor allem zunächst um die Internetanbindung und anschließend, um Bildgebungs- sowie Sensoriksysteme zu erweitern, damit weitere telemedizinische Anwendungsformen genutzt werden können (ID18; ID22; Lupton und Maslen 2017).

Die Durchführung der Studie weist Limitationen auf. Die Befragungsteilnehmer sind ausschließlich in der Intensivmedizin tätig. Werden die Stichprobenteilnehmer detaillierter betrachtet, so wird ein Überhang an Konsilgebern deutlich. Um einen besseren Eindruck der technologischen Schwierigkeiten der Konsilnehmer zu erhalten, sollten diese zukünftig stärker einbezogen werden. Weiterhin sind für die Implementierung und Nutzung einer Technologie noch andere Stakeholder wie das weitere klinische und technische Personal sowie die Klinikleitung und -verwaltung verantwortlich (Nothacker et al. 2019). Diese Stakeholder besitzen unterschiedliche Interessen. Während für das klinische Personal und die Ärzte die Behandlungsqualität im Fokus steht, berücksichtigt die Klinikleitung und -verwaltung wirtschaftliche Faktoren wie die Behandlungseffizienz (Nothacker et al. 2019). Diese mehrdimensionalen Perspektiven und Faktoren wurden im Rahmen dieser Studie nicht beachtet. Schließlich beschränkt sich diese Studie auf Kliniken in Nordrhein-Westfalen. Das deutsche Gesundheitssystem unterscheidet sich im internationalen Vergleich durch ein geringeren Digitalisierungsgrad (ID18). So existieren in anderen Ländern durch eine verbesserte Infrastruktur vielfältigere Anwendungsmöglichkeiten für Telekonsile wie zum Beispiel Echtzeitbefunde und -analysen (Forge und Vu 2020).

Zusammengefasst zeigen die Telekonsile im Rahmen dieser Studie, wie mit niederschwelligen, aktuell verfügbaren, Möglichkeiten die Behandlungsqualität für die Covid-19-Patienten erhöht, Expertenwissen regional verfügbar und ein höherer Behandlungsstandard etabliert werden kann. Besonders Expertenkonsile mit und ohne Fallbezug können aktuell bereits gut umgesetzt werden, während für Rekonsile teilweise noch eine verbesserte Internetanbindung und mehr Gerätschaften notwendig sind. Unsere Studie zeigt eine große Bandbreite in der Technologiebereitschaft der Krankenhäuser. Während manche Ärzte noch das Telefon verwenden und Patientenakten in Papierform pflegen, nutzen andere Kliniken bereits heute PDMS. Um in Zukunft die Telekonsile auszuweiten und weitere Anwendungsformen, die in Tab. 1 und in der Diskussion dargestellt werden, zu nutzen, muss die technologische Verfügbarkeit im Krankenhaus noch weiter verbessert werden. Die, in der Diskussion dargestellten, erweiterten Szenarien zeigen Entwicklungsformen der Telekonsile, die in Zukunft für ein breiteres Spektrum von Krankheitsbildern eingesetzt werden können und indikationsunabhängig Optimierungsmöglichkeiten bieten. Die zukünftige Forschung soll daran anknüpfend evaluieren, welche Faktoren für die Nutzung von Telekonsilen aus Sicht der verschiedenen Stakeholder relevant sind und so die Behandlung von Covid-19 als auch von anderen Krankheitsbildern sinnvoll ergänzen. Diese Erkenntnisse tragen somit zur verbesserten Integration und Implementierung neuer Technologien des Wissensaustauschs in jeglichen Bereichen des tertiären Sektors bei.
